# Immune Dysfunction in Polycystic Ovary Syndrome

**DOI:** 10.4049/immunohorizons.2200033

**Published:** 2023-05-17

**Authors:** Soma Banerjee, Laura G. Cooney, Aleksandar K. Stanic

**Affiliations:** * Division of Reproductive Sciences, Department of Obstetrics and Gynecology, University of Wisconsin–Madison, Madison, WI; † Division of Reproductive Endocrinology and Infertility, Department of Obstetrics and Gynecology, University of Wisconsin–Madison

## Abstract

Polycystic ovary syndrome (PCOS) is the most common endocrine disorder in reproductive-aged individuals with ovaries. It is associated with anovulation and increased risk to fertility and metabolic, cardiovascular, and psychological health. The pathophysiology of PCOS is still inadequately understood, although there is evidence of persistent low-grade inflammation, which correlates with associated visceral obesity. Elevated proinflammatory cytokine markers and altered immune cells have been reported in PCOS and raise the possibility that immune factors contribute to ovulatory dysfunction. Because normal ovulation is modulated by immune cells and cytokines in the ovarian microenvironment, the endocrine and metabolic abnormalities associated with PCOS orchestrate the accompanying adverse effects on ovulation and implantation. This review evaluates the current literature on the relationship between PCOS and immune abnormalities, with a focus on emerging research in the field.

## Introduction

Polycystic ovary syndrome (PCOS) (1) is the most common endocrine disorder in reproductive-aged individuals with ovaries, with a prevalence of 6–12% worldwide (2). PCOS is associated with anovulation and increased risk to reproductive, metabolic, cardiovascular, and psychological health (3–6). Rotterdam diagnostic criteria (2003) (1) define PCOS as the presence of two of three of the following: (1) clinical or biochemical hyperandrogenism, (2) oligomenorrhea, and (3) polycystic ovaries on ultrasound. Accompanying metabolic dysfunctions include insulin resistance, hyperinsulinemia, obesity, and hyperlipidemia. The pathophysiology of PCOS is still incompletely understood, although there is growing evidence of persistent low-grade inflammation beyond endocrine effects (7–9).

**FIGURE 1.**
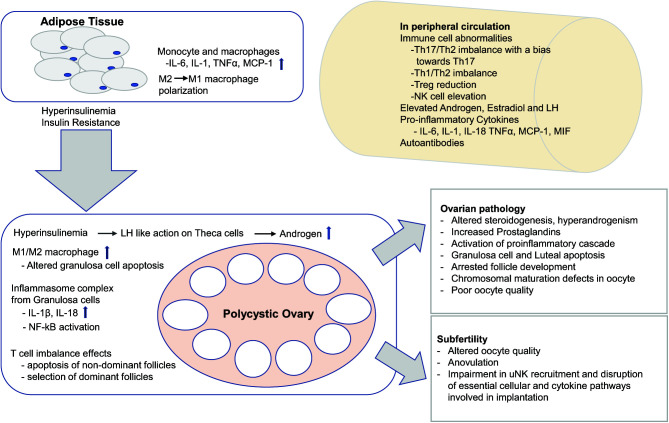
Inflammatory mediators involved in the pathophysiology of PCOS. Visceral adipocytes induce insulin activity, leading to impaired glucose tolerance, hyperinsulinemia, and insulin resistance. Hyperinsulinemia mimics the trophic action of LH in theca cells, increasing androgen production. Lastly, adipose tissue is rich in active immune cells (macrophages, monocytes) that secrete cytokines (IL-6, TNF-α, IL-1, MCP-1) associated with the inflammatory cascade in PCOS. The polarization of visceral adipose macrophages from M2 to M1 exacerbates the proinflammatory cytokine release from adipocytes. Peripheral immune cell abnormalities include a Th1 and Th17 bias, Treg cell reduction, and NK cell elevation. Notably, elevated IL-1β and IL-18 is seen in follicular fluid of patients with PCOS with evidence of NF-κB pathway activation and NLRP3 inflammasome formation in ovarian granulosa cells. The associated ovarian pathology includes altered steroidogenesis, hyperandrogenism, granulosa cell and luteal apoptosis, arrested follicular development, chromosomal maturation defects in oocytes, and poor oocyte quality. Impairment in uNK cell recruitment and disruption of essential cellular and cytokine pathways involved in implantation, altered oocyte quality, and anovulation are responsible for the resultant subfertility associated with PCOS.

The systemic (peripheral blood) proinflammatory bias in PCOS has been established in several studies, including elevated levels of C-reactive protein (10–13), IL-18 (14, 15), MCP-1 (16, 17), and MIP-1α (16) in plasma. In searching for the source of these cytokines, elevated circulatory total leukocyte count and leukocyte subset distribution (lymphocytes, neutrophils, NK cells) changes have been documented in PCOS (18, 19). Cellular and cytokine alterations are significant because normal immune system function is necessary for an appropriate ovulatory process. Within the ovary, immune cells secrete local cytokine mediators that promote follicle development, oocyte maturation, timely rupture of follicles, angiogenesis, corpus luteum development, and luteal demise (20), all of which may be altered in PCOS (Fig. 1). This review evaluates the accumulating literature on the significance of these cells and their secretory products for PCOS pathology, with a focus on emerging research in the field.

## Life-Course Stage, Early-Life Origins, and Genetics of PCOS

PCOS usually manifests during adolescence with maturation of the hypothalamic-pituitary-gonadal axis, although it may originate in very early development, even in intrauterine life (21). On the basis of animal models, it has been suggested that in utero androgen exposure may be associated with PCOS-like features in exposed progeny (22, 23). Nonhuman primate studies have shown that female rhesus monkeys exposed to high levels of testosterone in utero develop metabolic, clinical, and biochemical features of PCOS as adults. Intrauterine exposure to testosterone from non-PCOS causes such as congenital adrenal hyperplasia also leads to a PCOS phenotype (anovulatory cycles, hyperandrogenism, luteinizing hormone [LH] hypersecretion, polycystic ovaries, and insulin resistance) despite the normalization of androgen excess with treatment (24).

Pregnant individuals with PCOS have higher concentrations of androgens, which may affect fetal programming of the next generation (25). Although circulating maternal androgens or fetal adrenal androgens should normally be rapidly converted to estrogens by the activity of the placental aromatase enzyme (21), hyperinsulinemia has been shown to inhibit aromatase activity in human cytotrophoblasts (26). Thus, female offspring may still be exposed to excess androgen. Last, PCOS pregnancy is associated with a higher incidence of small for gestational age newborns (27), an independent risk factor for metabolic dysfunction and insulin resistance (28). These in turn can result in functional hyperandrogenism, premature pubarche, and PCOS during adolescence (29, 30). Daughters of women with PCOS have elevated anti-Müllerian hormone (AMH) in infancy, early childhood, and prepuberty, suggesting an ovarian component to the heritability of PCOS pathology (31–33).

Family and twin studies indicate an inherited component to PCOS (34). Biochemical and genotype data have identified several risk genes associated with the different reproductive and metabolic phenotypes of PCOS by genome-wide association study (35). Particularly pertinent are genes that regulate gonadotropin secretion and action or ovarian function (FSHB, FSHR, LHCGR, AMH, AMHR2) (36), AMH variants (37, 38), ovarian and adrenal steroidogenesis (CYP11a, CYP21, CYP17, CYP19) (39), and androgen function (17βHSD, AR, SHBG) (39). Interestingly, meta-analysis of several studies indicates that PCOS risk is positively associated with specific TNF-α and IL-6 gene polymorphisms (40), indicating that genetic predisposition may influence immune dysfunction in this condition.

Epigenetic modifications may also contribute to PCOS susceptibility with associated reproductive and metabolic dysfunction. Genome-wide epigenetic profiling of DNA methylation, histone modifications, and noncoding RNAs has been performed in tissues derived from patients with PCOS (35). Subjects with PCOS have dysfunctional adipose tissue with larger adipocytes, lower serum adiponectin levels, and lower adipose tissue lipoprotein lipase activity (41). In addition, a study that compared global DNA methylation patterns in s.c. adipose tissue of subjects with PCOS and control subjects identified a total of 440 sites with differential CpG methylation (42). The differentially expressed genes in PCOS correlated with the transcriptional regulation of pathways involved in inflammation, adipogenesis, energy metabolism, sex hormone metabolism, and glucose regulation.

The role of epigenetic modifications has also been demonstrated recently in immune cell subsets of patients with PCOS. The DNA methylome of immune cells in individuals with PCOS demonstrates genome-wide lower global DNA methylation in monocytes and T helper (Th), cytotoxic T (Tc), and B cells (43). Specific genome-wide DNA methylation analysis showed that hypomethylation of CpG sites in PCOS correlated with elevated AMH and free testosterone in Th cells only. Gene ontology analysis of the genes harboring differentially methylated cytosines identified genes related to T cell function, as well as reproductive function (pregnancy, prolactin response, ovarian follicle development, progesterone receptor signaling pathway, male sex determination, and response to steroid hormones).

## Environmental Factors in Susceptibility and Onset of PCOS

Intrauterine fetal programming in PCOS may also be attributed to prenatal exposure to environmental toxins, especially known endocrine-disrupting chemicals (44). Bisphenol acetate is an endocrine-disrupting chemical that acts as both agonist and antagonist at the estrogen receptors, depending on cellular context, and binds androgen receptors (ARs) as well. Rat models demonstrate that exposure to high doses of bisphenol acetate during the neonatal period results in a PCOS-like phenotype in adulthood, including altered gonadotropin-releasing hormone pulsatility, elevated androgens, and ovarian cysts (45). Taken together, inherited and acquired genetic changes as well as environmental exposures appear to link reproductive molecular pathways and immune pathways in the pathogenesis of PCOS.

## The Role of Obesity and Metabolic Syndrome in the Chronic Inflammatory Pathophysiology of PCOS

Central type (visceral or abdominal) obesity is highly prevalent in PCOS (54%; 95% confidence interval, 43–62%) and promotes metabolic, endocrine, and immune dysfunction in patients with PCOS. Central fat is strongly linked to insulin resistance and type 2 diabetes (46, 47), because visceral adipocytes induce defective insulin activity (48) and convert cortisone to metabolically active cortisol (49), leading to impaired glucose tolerance, hyperinsulinemia, and insulin resistance. Adipose tissue can also contribute to the PCOS-associated androgen excess by converting Δ-4 androstenedione to the more potent androgen testosterone via 17-β hydroxysteroid dehydrogenase enzyme (49).

The hyperandrogenism in PCOS is derived primarily from ovaries, with a contribution from adrenal glands as well (50, 51). Hyperinsulinemia augments the trophic action of LH in theca cells, thereby activating the side chain cleavage enzyme in the 17-hydroxylase and 17,20-lyase enzyme complex and increasing androgen production (52, 53).

Endocrine and metabolic effects of adipose tissue on PCOS pathology are further amplified by the abundance of local active immune cells (macrophages, monocytes) (54, 55). These fat-resident immune cells secrete a variety of cytokines (IL-6, TNF-α, IL-1, MCP-1) contributing to the PCOS inflammatory cascade (56).

## Cytokine Mediators of Chronic Inflammation in PCOS

Cytokines are molecules that are involved in many physiological processes, including the regulation of immune and inflammatory responses. They are often produced by local tissues and control the duration and strength of the immune response. Over- or underproduction of cytokines can contribute to the pathophysiology of multiple diseases. In the reproductive system, cytokines are also involved in the normal pathway of follicle recruitment and ovulation, and derangements can impact egg quality. In addition, many of the comorbidities associated with PCOS, including obesity and diabetes, are associated with abnormal cytokine production or signaling. Thus, it has been hypothesized that some of the pathophysiology of PCOS may be involved with abnormal cytokine production or signaling.

### IL-6 and TNF-α

IL-6, which is produced from mononuclear cells and adipocytes, is a major orchestrator of a chronic proinflammatory state. It regulates hepatic C-reactive protein secretion that is induced by insulin resistance, dyslipidemia, and central obesity (57). Studies in the general population have shown a positive correlation between elevated IL-6, obesity, insulin resistance, and hyperandrogenism (56), which are all comorbidities associated with PCOS. Individuals with PCOS also have elevated IL-6 (58, 59); however, it is unclear if it is explained solely by obesity or if PCOS itself plays a role, because backward regression analysis demonstrated a stronger association of IL-6 elevation with PCOS independent of obesity and sleep apnea (59).

TNF-α is a proinflammatory cytokine with a multitude of effects in many tissues, including muscles, adipose tissues, macrophages, and ovaries. TNF-α binds to its receptor (TNFR1), followed by caspase-8 and caspase-3 activation, inducing downstream inhibitor of NF-κB phosphorylation and degradation, releasing NF-κB. Subsequently, NF-κB translocates to the nucleus, where it activates genes responsible for immune and inflammatory responses (60). In normal ovaries, TNF-α and IL-6 serve important roles in ovarian steroidogenesis (61), granulosa and luteal cell apoptosis (62), follicular atresia, a luteolytic effect by inducing local prostaglandin (PG) production (62), and an effect on oocyte quality (63, 64). Animal model studies demonstrate theca-interstitial cell proliferation in rats (61), as well as chromosome maturation defects and abnormal chromosomal alignment in porcine oocytes (63) exposed to high levels of TNF-α. Interestingly, elevations of TNF-α and IL-6 are found together in the serum and follicular fluid of women with PCOS (63, 65). TNF-α can induce systemic insulin resistance by inhibiting insulin receptor tyrosine kinase in muscle and fat, suggesting a possible role in metabolic syndrome seen in PCOS (60). In addition, a meta-analysis showed an association between TNF-α and IL-6 gene polymorphisms and the risk of PCOS (40), suggesting that immunological dysfunction could predispose individuals to the ovulatory abnormalities initiating PCOS. Taken together, these studies present the tantalizing but still unproven hypothesis that IL-6 and TNF-α in conjunction directly modulate insulin resistance, androgen excess, and ovulatory dysfunction in PCOS.

### IL-1β and IL-18

IL-1β and IL-18 are members of the proinflammatory IL-1 cytokine superfamily produced primarily from monocytes and macrophages. IL-1β has been demonstrated to induce follicular maturation and rupture in various animal models by regulating granulosa and theca cell steroidogenesis, ovarian protease and PG production, and oocyte nuclear maturation (66–69). IL-1β and IL-18 are both elevated in the follicular fluid of patients with PCOS (15, 70). Their roles in the inflammasome complex and the pathophysiology of PCOS are emerging and require further elucidation (70).

### MCP-1 and migration inhibitory factor (MIF)

Chemokines MCP-1 and MIF are key innate immune mediators regulating monocyte/macrophage trafficking in a variety of tissues (71). MCP-1 is a potent recruitment agent for monocytes and macrophages that is produced in adipose tissue and ovaries by endothelial cells, fibroblasts, and immune cells, including neutrophils and monocytes/macrophages. It regulates several ovulatory processes in normal ovarian physiology (72) and is elevated in serum and follicular fluid in PCOS (73–75).

MIF, a macrophage product, is produced during normal follicular development and ovulation in animal models (76). Conversely, MIF is elevated in PCOS (17), obesity, insulin resistance, and pancreatic β-cell defects (77–79).

These changes in local chemokines could alter ovarian follicle-resident macrophages, in turn disrupting normal ovulatory function (progressive infiltration of lymphocytes and macrophages, tissue remodeling, release of ovum, luteal formation and regression); however, the mechanism driving this pathology is still poorly understood.

## PCOS and Immune Cell Subsets

In conjunction with cytokine alterations, immune cell composition is also disturbed in PCOS pathology, especially when considering that normal follicular development and ovulation are themselves modulated by a carefully controlled inflammatory process.

### Macrophages and dendritic cells

Macrophages produce many cytokine/chemokine components of the proinflammatory cascade that characterizes metabolic and ovarian dysfunction in PCOS. The accumulation of macrophages in visceral adipose tissue results in the release of IL-1, IL-6, IL-10, IL-12, NO, and TNF-α into the circulation (54, 57). Macrophage density in mammalian ovaries varies on the basis of cellular region and hormone levels. Macrophages are abundant in human follicular fluid, are low in the thecal layer of developing follicles, peak during the preovulatory phase, and remain high in the regressing corpus luteum and atretic follicles (80, 81). Animal model studies indicate that macrophages may be indispensable for the maintenance of follicle and corpus luteum structure and integrity. Diphtheria toxin–driven conditional depletion of ovarian macrophages in adult CD11b diphtheria toxin receptor mice produces widespread hemorrhage and cell death in developing follicles (82). These mice also exhibit abnormal corpora lutea, with elevated inflammation and apoptosis genes (PTGS2, HIFL1A), along with diminished expression of steroidogenesis genes (STAR, CYP11A1, and HSD3B1) (83). PCOS-associated follicular dysfunction may be critically linked to altered macrophage function.

Homocysteine, an amino acid associated with insulin resistance and implicated in the development of cardiovascular disease, is elevated in individuals with PCOS (84). A dehydroepiandrosterone sulfate–induced PCOS mouse model has been created to evaluate changes in macrophage polarization in the setting of hyperhomocysteinemia to mimic the physiology of PCOS. In this model, macrophage polarization in the visceral adipose tissue changes from an anti-inflammatory M2 state to a proinflammatory M1 state in mice with elevated homocysteine levels (85). Notably, in this model, hyperhomocysteinemia leads to elevations in TNF-α, IL-6, and MCP-1 and exaggerates insulin resistance (85), risk factors for cardiovascular diseases observed in PCOS.

In a different dihydrotestosterone-induced PCOS model in rats, follicular dynamics are altered with increased M1/M2 macrophage ratio (proinflammatory) in antral and preovulatory follicles (86). Androgen-induced granulosa cell apoptosis was also noted in this model, suggesting a possible mechanism for the follicular arrest seen in PCOS.

### T lymphocytes

T lymphocytes are central to immune regulation, and abnormalities in their number and function have been studied extensively in PCOS. Broadly, T cells are classified into Th cells, Tc cells, and regulatory T (Treg) cells. T cells participate in normal ovarian functions, including ovarian homeostasis, apoptosis of nondominant follicles, and selection of dominant follicles (87).

#### T-helper cells

Th (CD4^+^) cells can be subdivided into different subsets on the basis of cytokines produced and the immune impact: (1) Th1 cells produce IL-2 and IFN-γ and drive cellular immunity; (2) Th2 cells produce IL-4, IL-5, and IL-13 and direct humoral immunity; and (3) Th17 cells produce proinflammatory cytokines IL-17A, IL-17F, and IL-21 associated with extracellular pathogen defense and autoimmunity. The interactions of these cytokines play vital roles in pregnancy and fertility. For example, a productive pregnancy involves a decidual Treg cell dominance over T effector Th1/Th2/Th17 responses, whereas dysfunction may contribute to recurrent spontaneous abortion and preeclampsia.

Similarly, Th17/Th2 ratio alterations with a bias toward Th17 and reduced Treg cell numbers may be a factor in the increased risk of autoimmunity (see *Treg cells* section below) with PCOS (88). Poor ovum quality and poor pregnancy outcomes are associated with altered Th1/Th2 balance in PCOS (89). Elevation of androgens and unopposed estradiol in PCOS also augment the secretion of T cell–modulating cytokines TNF-α, IL-6, IL-12, and IFN-γ (90).

#### Cytotoxic T cells

Tc (CD8^+^) cells are the primary effector cells of cellular immunity that eliminate infected or malignantly transformed cells. The proportion of CD3^+^CD8^+^ T lymphocytes is significantly reduced in PCOS follicular fluid alongside increased PD-1 expression proportional to serum estradiol level (91). Interestingly, in PCOS, the CD4^+^CD28^null^ Tc cells are elevated (92), a cell phenotype with proinflammatory functions, producing high levels of IFN-γ, TNF-α, and IL-2, all of which are cardiovascular disease risk factors (93), adding to the association between inflammation and PCOS.

#### Treg cells

Several studies implicate Treg cell abnormalities in PCOS (88, 94). Treg cells express Foxp3, IL-10, and TGF-β and account for ∼1–2% of the total CD4^+^ T cells, helping prevent autoimmune diseases by restraining proliferation of effector T cells and inflammatory cytokine production. In a single study, among subjects with PCOS, the proportion of Treg cells was found to be low, along with increased Th1/Th2 and Th17/Th2 ratio (88). Moreover, Treg cell–induced immune-suppressing cytokines IL-10 and TGF-β were low as well, whereas proinflammatory cytokines IL-6, IL-17, and IL-23 were elevated, in PCOS (94). Because pregnancy complications such as recurrent pregnancy loss and preeclampsia are linked to reduced Treg cell function (95, 96), alterations of Treg cells in pregnant patients with PCOS could be a significant contributor to adverse pregnancy outcomes seen in this syndrome. Additionally, Treg cell dysfunction may be the reason underlying the strong association of PCOS with circulating autoantibodies (antinuclear Abs, antithyroid Abs, islet cell Abs, anti-histone, anti-ovarian, and others) and autoimmune thyroiditis, type 2 diabetes mellitus (97).

### B cells

A single study indicated that the proportion and activity of autoantibody-secreting CD19^+^ B cells are significantly increased in the peripheral blood of subjects with PCOS (98). Most strikingly, in a dehydroepiandrosterone-induced PCOS mouse model, depletion of B cells by CD19 Ab prevented the development of a polycystic ovarian phenotype, and those mice retained normal body weight. Additionally, expression levels of fibrosis factors (collagen type III α1 chain and connective tissue growth factor) in mice ovaries were attenuated with CD19 Ab pretreatment. These data form an exciting preliminary indication that immune cell modulation is a possible avenue to modify PCOS pathophysiology and establish a mechanistic link between them. However, extensive examination of the B cell and innate lymphocyte (NK cell and ILC, respectively) function and relationship to PCOS pathophysiology in humans and animal models is currently lacking.

### ILCs and NK cells

Subfertility is a key feature of PCOS pathology, with recent studies suggesting a link between NK cell dysfunction and this clinical presentation. The regulation of NK cells in the peripheral blood and endometrium has been implicated in myriad reproductive disorders, including recurrent/spontaneous abortions, implantation failure, infertility, and preeclampsia (99). Steroid-dependent changes in uterine NK cells (uNK cells; CD3^−^/CD56^bright^CD16^−^) facilitate implantation and pregnancy maintenance (99). CXCL10, IL-15, and IL-18 recruit CD16^−^ NK cells from the circulation to the endometrium and/or stimulate proliferation of resident uNK cells (100, 101). In PCOS, circulating NK cells are elevated, and endometrial CXCL10, IL-15, IL-18, and IL-12A are reduced (102). There is accompanying reduction of uNK cells and elevation of CD3^+^ lymphocytes (102), as well as CD68^+^ macrophages, CD163^+^ M2 macrophages, CD1a^+^ interstitial dendritic cells, CD83^+^ myeloid dendritic cells, and CD8^+^ T cells (103), in the endometrium of infertile individuals with PCOS. Hence, rather than simply being a consequence of anovulation, at least in some individuals, PCOS-associated infertility may be critically dependent on impairment in uNK cell recruitment and disruption of essential cellular and cytokine pathways involved in implantation.

Establishment of a normal maternal-fetal decidual interface is mediated by the killer cell Ig-like receptors (KIRs) on the surface of NK cells that modulate the activation and function of these cells upon interacting with HLA class I ligands. A frequency distribution study of KIR and HLA class I genotypes shows that higher frequency of hypofunctional KIR3DS1-Bw4 and homozygous KIR2DS4-del and lower frequency of KIR2DS4-full are linked to PCOS susceptibility (104). Because KIR3DS1 and KIR2DS4 are activating receptors (105, 106), diminished NK cell activation may provide the mechanistic link between PCOS susceptibility alleles and observed adverse fertility outcomes. These genetic data are additional tantalizing evidence that immune aberration not only is associated with but also may be a direct predisposing factor in the pathophysiology of PCOS.

The human decidua contains all ILC groups: ILC1, ILC2, ILC3, and lymphoid tissue inducer-like cells (107). ILCs have been shown to predominate and exert their function primarily in mucosal tissues and participate in decidual remodeling and VEGF production at the fetal-maternal interface (107, 108). Thus, because ILCs are very important in normal pregnancy, and because these immune populations are poorly understood in PCOS, this is a very fertile area of future investigation.

## Future Directions and Emerging Research Areas

### Androgen signaling effects on immune dysfunction in PCOS

Excess production of androgens, including ovarian testosterone, androstenedione, and adrenal dehydroepiandrosterone sulfate, is a key feature of PCOS (52). AR signaling has been shown to affect many immune cell types directly and indirectly and is thought to be largely immunosuppressive (109).

AR is expressed in most neutrophil lineages, from proliferative precursors to mature neutrophils (110). AR-knockout mice have reduced neutrophil counts, reduced precursor proliferation, delayed maturation, and increased susceptibility to apoptosis (111). Testosterone also modulates human macrophage function by suppressing the expression of proinflammatory TNF-α and IL-1β in vitro (112). Androgen treatment creates a shift toward a Th2 immune response by enhanced production of IL-10 by murine CD4^+^ T cells (113). Finally, a study demonstrated a sex-specific difference in CD4^+^CD25^+^Foxp3^+^ Treg cell distribution between men and women. The Foxp3^+^ Treg cell population is higher in women than in men, and dihydrotestosterone treatment induces an ovulatory phase proliferation of these cells in women (114).

These findings indicate that testosterone generates an overall immunosuppressive effect. Yet, despite elevated circulating androgens, PCOS is characterized by a predominantly proinflammatory bias, indicating that further study is required to decipher the immune effects of hyperandrogenism in PCOS.

### Inflammasome complex and pathophysiology of PCOS

The process of normal follicular development and ovulation is mediated by a carefully controlled inflammatory process involving PGs, inflammatory cytokines, chemokines, and activation of the inflammasome complex orchestrated by and acting upon follicular immune cells. The inflammasome has three components: a sensor, an adaptor, and an effector (115). The sensors, including TLRs, recognize different pathogen-related molecular patterns, triggering the recruitment of the adaptor protein adipose tissue–derived stem cell protein that initiates the effector component, caspase-1. Nod-like receptor (NLR) family pyrin domain-containing 3 (NLRP3) is an intracellular sensor that recognizes endogenous damage-associated molecular patterns and forms a cytoplasmic complex called the “NLRP3 inflammasome” with adipose tissue–derived stem cells and pro-caspase-1, which regulates the maturation and secretion of IL-1β and IL-18, in an autocrine, self-amplifying loop. Subsequently, NF-κB is activated by phosphorylation and degradation of inhibitor of NF-κB, release and transport into the nucleus, and induction of immune response genes of the inflammatory cascade (e.g., TNF-α, IFN-γ, IL-6, VEGF).

IL-1β and IL-18 were recently detected at elevated levels within follicular fluid of patients with PCOS with evidence of NF-κB pathway activation and NLRP3 inflammasome formation in ovarian granulosa cells (70, 116). Furthermore, the granulosa cell carcinoma cell line KGN activated with LPS or stimulated by follicular fluid from patients with PCOS produced NLRP3 inflammasomes (70). Evidence of mitochondrial structural and functional damage was observed in granulosa cells, indicating possibilities of mechanisms such as oxidative stress, altered cellular metabolism, and impaired cell proliferation in the pathogenesis of PCOS (70). Taken together, this evidence implies that follicular environment inflammatory stress in PCOS may be an initiating factor for the associated ovulatory dysfunction, although more conclusive investigation is necessary.

### Single-cell technologies and PCOS

The complex cellular environment of the ovarian follicle, endometrium, and decidua, as well as pivotal cell-to-cell communication in these reproductive tissues, requires collection of comprehensive RNA, protein, and signaling data at the level of individual cells (117). Infections, cancer, and abnormal reproductive physiology, including preterm labor, are increasingly studied at a single-cell level and promise useful targets for medical intervention. Single-cell RNA sequencing of human oocytes affected by PCOS at germinal vesicle (GV), metaphase I, and metaphase II stages demonstrates upregulated genes at the GV stage affecting mitochondrial function, such as COX6B1, COX8A, COX4l1, and NDUFB9 (118). These same genes were activated at the metaphase II stage in healthy oocytes, suggesting that some mitochondrial functions may be prematurely activated at the GV stage in PCOS, contributing to a decline in oocyte quality. Much less is known about other cellular constituents of the developing follicle at the single-cell level, and, given the important role of immune cells in ovarian function and PCOS, this is a key direction of future study.

The endocrine and metabolic abnormalities seen in PCOS induce a chronic inflammatory process. The link between visceral adipocytes and the ovarian microenvironment may be through inflammatory cytokines and immune cells in the circulation; however, the details of this process are yet to be discovered. Normal follicular development and ovulation are themselves modulated by a carefully controlled inflammatory process that is altered in PCOS. The inflammatory stress initiated in PCOS is exemplified by the formation of inflammasome complexes in granulosa cells. It is thus a distinct possibility, yet unproven, that immune dysfunction directly results in ovulatory dysfunction. The roles of the inflammatory cytokines (IL-6, TNF-α, IL-1, IL-18, MCP-1), Th1 and Th17 bias, and reduction of Treg cell and macrophage polarization in the pathogenesis of PCOS are better understood than other immune proteins and cells. Very limited information exists regarding NK cells and ILCs in PCOS, which is a particularly important research direction, given their role in maintaining normal pregnancy/placentation and in light of infertility associated with PCOS. Emerging research is required to elucidate the role of androgen excess and immune abnormalities, and it is hoped that innovative research modalities at a single-cell level will provide important insights into these avenues.
